# Hemostasis as soon as possible? The role of the time to angioembolization in the management of pelvic fracture

**DOI:** 10.1186/s13017-019-0248-z

**Published:** 2019-06-13

**Authors:** Chang-Hua Chou, Yu-Tung Wu, Chih-Yuan Fu, Chien-Hung Liao, Shang-Yu Wang, Francesco Bajani, Chi-Hsun Hsieh

**Affiliations:** Department of Trauma and Emergency Surgery, Chang Gung Memorial Hospital, Chang Gung University, 5, Fu-Hsing Street, Kwei Shan Township, Taoyuan, Taiwan

**Keywords:** Pelvic fracture, TAE, Length of stay, Timing, Angioembolization

## Abstract

**Introduction:**

While transcatheter arterial embolization (TAE) is an effective way to control arterial bleeding associated with pelvic fracture, delayed TAE may increase mortality risk. The purpose of the current study was to determine how time to TAE affects outcomes in patients with pelvic fracture in the emergency department.

**Methods:**

From January 2014 to December 2016, the trauma registry and medical records of patients with pelvic fracture who underwent TAE were retrospectively reviewed. The relationship between the time to TAE and patient outcomes was evaluated. The characteristics of surviving and deceased patients were also compared to search for prognostic factors affecting survival.

**Results:**

Eighty-four patients were enrolled in the current study. Among patients with pelvic fracture who underwent TAE, the overall mortality rate was 16.7%. There were positive relationships between the time to TAE and the requirement for blood transfusion and between the time to TAE and intensive care unit (ICU) length of stay (LOS). Nonsurviving patients were significantly older (57.4 ± 23.3 vs. 42.7 ± 19.3 years old, *p* = 0.014) and had higher injury severity scores (ISSs) (36.4 ± 11.9 vs. 23.9 ± 10.9, *p* < 0.001) than were observed in surviving patients. There was no significant difference in the time to TAE between nonsurviving and surviving patients (76.9 ± 47.9 vs. 59.0 ± 29.3 min, *p* = 0.068). The multivariate logistic regression analysis showed that ISS and age served as independent risk factors for mortality. Every one unit increase in ISS or age resulted in a 1.154- or 1.140-fold increase in mortality, respectively (*p* = 0.033 and 0.005, respectively). However, the time to TAE serves as an independent factor for ICU LOS (*p* = 0.015).

**Conclusion:**

In pelvic fracture patients who require TAE for hemostasis, longer time to TAE may cause harm. An early hemorrhage control is suggested.

## Introduction

According to previously published statistics, pelvic fractures constitute approximately 3% of skeletal injuries, and the most common causes of pelvic fractures include motor vehicle collisions, motorcycle collisions, auto-pedestrian collisions, and falls from heights [1–3]. Due to hemorrhage, 5% to 20% of patients have unstable hemodynamics, and hemorrhage-related mortality rates as high as 40% have been reported [4]. Resuscitation with blood product transfusion and temporary mechanical stabilization are necessary to combat pelvic fracture hemorrhage [5–8]. However, pelvic arterial bleeding accounts for up to 15% of bleeding associated with pelvic fractures; this type of bleeding is more threatening than venous bleeding [2, 9, 10]. The most often identified sources of arterial bleeding are the internal iliac artery (IIA) and its branches; patients with pelvic arterial bleeding usually present with systolic blood pressure (SBP) less than 90 mmHg, need more than 2000 mL of fluid resuscitation, and receive more than 4 units of blood transfusion within 24 h [2, 11–13]. Transcatheter arterial embolization (TAE) is an effective way to aggressively manage arterial bleeding and has a success rate higher than 85% [5, 10, 14–21].

Unfortunately, although the success rate of TAE is high, the mortality rates reported in patients receiving TAE range from 16% to 50% among previous studies [11, 22]. Deaths usually result from associated injuries and delayed TAE [7, 19, 22]. While a patient waits for TAE, ongoing hemorrhage may increase his or her mortality risk over time. Performing TAE less than 3 h after admission leads to better outcomes, but many previous studies have shown that it is difficult to achieve this goal [6, 7, 19, 22–25].

We hypothesize that even when the time from emergency department (ED) admission to TAE is significantly less than 3 h, earlier TAE can still result in better outcomes. The purpose of the current study was to determine how time to TAE affects outcomes in patients with pelvic fracture in the ED.

## Materials and methods

As a level-I trauma center in Taiwan, in our institution, the attending-level trauma surgeons are responsible for the initial survey and treatment of patients with pelvic injuries in the ED. Adequate and timely evaluation and management are provided for all traumatized patients. Patients with multiple traumas or hemodynamic instability have higher priority, and patients with pelvic fracture who need TAE are promptly identified. In our institution, interventional radiologists and the equipment required for TAE are available 24 h a day, 7 days a week. Thus, the time from ED admission to TAE can be less than 1 h. During the angiographic examination, the abdominal aorta, lumbar arteries, bilateral common iliac arteries, bilateral external iliac arteries, and bilateral internal iliac arteries were routinely evaluated. Embolization would be performed proximal to sites of arterial extravasation. To prevent long-term complications related to embolization, the application of Gelfoam® slurry, a temporary embolic agent that is likely biodegraded in 7–21 days and allows recanalization, was used for the distal blind embolization [26]. However, in certain rare patients whose hemorrhage could not be controlled with Gelfoam embolization, a coil would be utilized for permanent embolization.

**Fig. 1:**
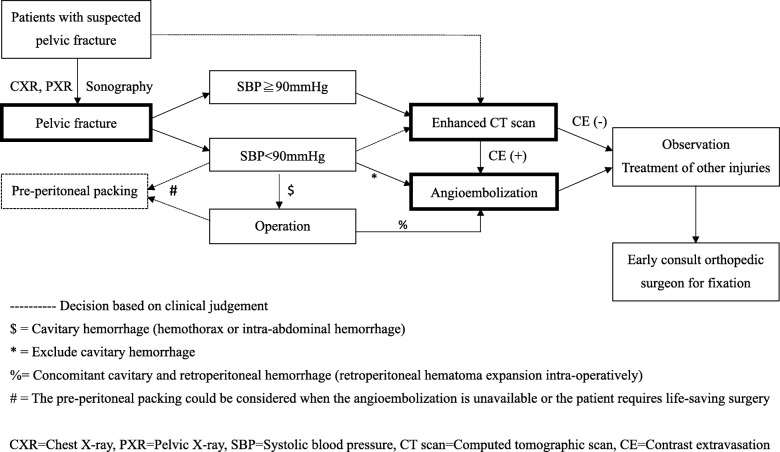
Pelvic fracture management algorithm

Patients with pelvic fractures without other extrapelvic injuries requiring emergent treatment are treated according to a previously published algorithm (Fig. 1) [27]. Patients with pelvic fractures received airway protection, fluid resuscitation, a pelvic circumferential compression device for unstable pelvic fracture, external hemorrhage control, and other necessary evaluation before TAE based on the Advanced Trauma Life Support (ATLS) guidelines [28]. According to the algorithm, TAE is indicated for patients with intrapelvic contrast extravasation on computed tomographic (CT) scan or unstable hemodynamics without other cavitary or external bleeding. The trauma registry and medical records of patients with pelvic fracture (International Classification of Diseases-9 code: 808) who were admitted to our institution and underwent TAE from January 2014 to December 2016 were retrospectively reviewed. Patients with pelvic fractures who were sent to TAE directly from the ED were enrolled in the current study. Patients who were younger than 18 years old, had out-of-hospital cardiac arrest (OHCA) without a response to resuscitation, died in the ED, or received TAE after treatment of other injuries [ex., thoracotomy, laparotomy, delayed hemorrhage during intensive care unit (ICU) observation] were excluded.

In this study, the characteristics of pelvic fracture patients who received TAE are described. The relationship between the time to TAE and patient outcomes was evaluated with regard for different aspects. The characteristics of surviving and deceased patients were also compared to search for the prognostic factors affecting survival.

### Statistics

Numerical data are presented as the means and standard deviations, and categorical data are reported as percentages. Student’s *t* test was used to compare numerical data, and the chi-square test was used to compare categorical data. Covariables with significance in univariate analysis were analyzed in a multivariate regression model. Multivariable logistic regression was used to evaluate independent risk factors for mortality in the patients in our study, and multivariable linear regression was used to evaluate factors that affected the ICU length of stay (LOS). All analyses were performed using IBM SPSS software (version 22.0, Chicago, IL, USA).

## Results

**Fig. 2:**
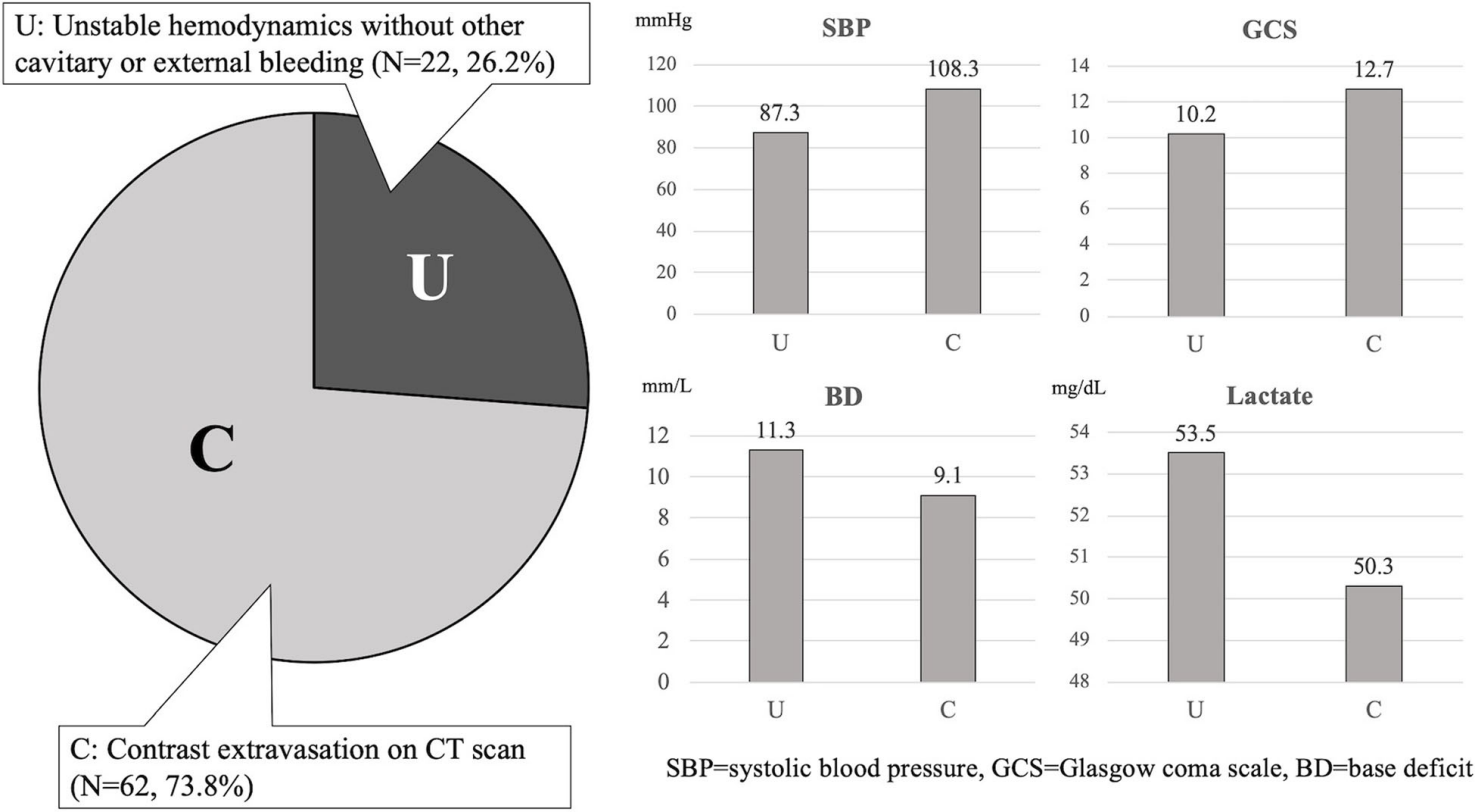
Indications for TAE and the characteristics of patients with different indications for TAE

According to the trauma registry databank maintained by our institution, a total of 609 patients with pelvic fractures who were admitted to our ED from January 2014 to December 2016 were evaluated. A total of 89 (14.6%) of these patients received TAE of the IIA system or external iliac artery (EIA) and its branches. Eighty-four of them met the inclusion criteria of the current study. The mean patient age was 45.2 ± 20.6 years old, and 43 were male (51.2%), while 41 were female (48.8%). The physical data of these patients showed that they had a mean SBP of 102.8 ± 34.9 mmHg, a mean Glasgow Coma Scale (GCS) of 12.1 ± 4.4, a mean base deficit (BD) of 9.8 ± 6.0 mm/L, and a mean lactate level of 51.4 ± 34.0 mg/dL. The mean injury severity score (ISS) was 26.0 ± 12.0, and the mean blood transfusion volume was 1047.6 ± 949.4 mL. Of these 84 patients, 62 (73.8%) received TAE because of contrast extravasation on CT scans, and the other 22 (26.2%) patients received TAE because of unstable hemodynamics without other cavitary or external bleeding (Fig. 2). The mean time to TAE was 62.0 ± 33.4 min, and the mean procedure time was 50 min (ranging from 30 to 140 min). Forty-seven patients (56.0%) received TAE less than or equal to 1 h after admission, and 37 patients (44.0%) received TAE more than 1 h after admission. The mean ICU LOS was 5.9 ± 5.9 days, and the mean hospital LOS was 22.2 ± 14.0 days. Fourteen patients died in the ICU or an ordinary ward, and the overall mortality rate was 16.7% (Table 1). Four (28.6%) of the 14 patients died of pelvic fracture-related hemorrhage, and ten (71.4%) died of other associated injuries, including respiratory failure, traumatic brain injury, sepsis, and traumatic pulmonary hemorrhage. Patients whose deaths were related to pelvic fracture-related hemorrhage died earlier than patients whose deaths were related to other associated injuries (2.7 ± 4.4 days vs. 22.9 ± 14.8 days).

**Table 1:** General demographics of the pelvic fracture patients who received angioembolization (*N* = 84)

Factors	No. (%) or Mean ± SD (*N* = 84)
Age (y/o)	45.2 ± 20.6
Sex	
Male	43 (51.2%)
Female	41 (48.8%)
SBP upon ED arrival (mmHg)	102.8 ± 34.9
GCS upon ED arrival	12.1 ± 4.4
ISS (score)	26.0 ± 12.0
Total blood transfusion (mL) in ED	1047.6 ± 949.4
Indications for TAE	
1. Contrast extravasation on CT scan (*N*, %)	62 (73.8%)
2. Unstable hemodynamics without other cavitary or external bleeding (*N*, %)	22 (26.2%)
Time to TAE	62.0 ± 33.4 min
≦ 1 h	47 (56.0%)
> 1 h	37 (44.0%)
Mean hospital length of stay (LOS)	22.2 ± 14.0 days
Mean ICU LOS	5.9 ± 5.9 days
Outcomes	
Survival	70 (83.3%)
Mortality	14 (16.7%)

The comparison between nonsurviving and surviving patients revealed that the nonsurviving patients were significantly older (57.4 ± 23.3 vs. 42.7 ± 19.3 years old, *p* = 0.014), had lower GCS scores upon arrival (7.6 ± 5.0 vs. 13.0 ± 3.7, *p* = 0.002), had a greater BD (13.5 ± 4.5 vs. 8.8 ± 6.1 mm/L, *p* = 0.005), had higher abbreviated injury scale (AIS) scores for the head/neck (2.1 ± 2.1 vs. 0.9 ± 1.5, *p* = 0.006), had higher AIS scores for the chest (2.8 ± 2.0 vs. 1.7 ± 1.7, *p* = 0.017), and had higher ISSs (36.4 ± 11.9 vs. 23.9 ± 10.9, *p* < 0.001). However, no significant differences in sex, blood transfusion volume, or the time to TAE (76.9 ± 47.9 vs. 59.0 ± 29.3 min, *p* = 0.068) were found between the nonsurviving and surviving patients (Table 2).

**Table 2:** Comparison of the characteristics between nonsurviving and surviving pelvic fracture patients who received angioembolization (*N* = 84)

	Nonsurvivors (*N* = 14)	Survivors (*N* = 70)	*p* value
Age (y/o)	57.4 ± 23.3	42.7 ± 19.3	0.014^a^
Male (%)	7 (50.0%)	36 (51.4%)	0.702^b^
ISS	36.4 ± 11.9	23.9 ± 10.9	< 0.001^a^
Blood transfusion (mL)	1214.3 ± 1086.9	1014.3 ± 924.6	0.475^a^
Time to TAE (min)	76.9 ± 47.9	59.0 ± 29.3	0.068^a^

^a^Student’s *T* test

^b^Chi-square test

**Fig. 3:**
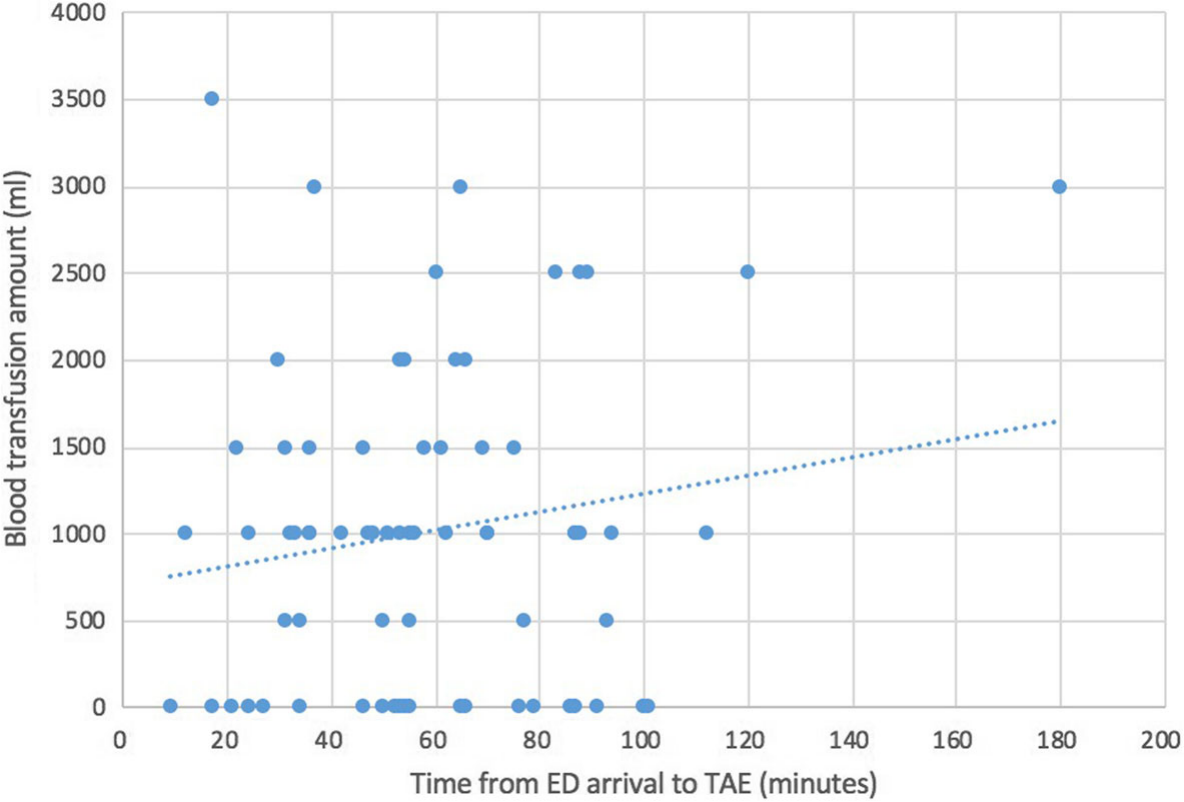
The relationship between the time to TAE and ICU length of stay

**Fig. 4:**
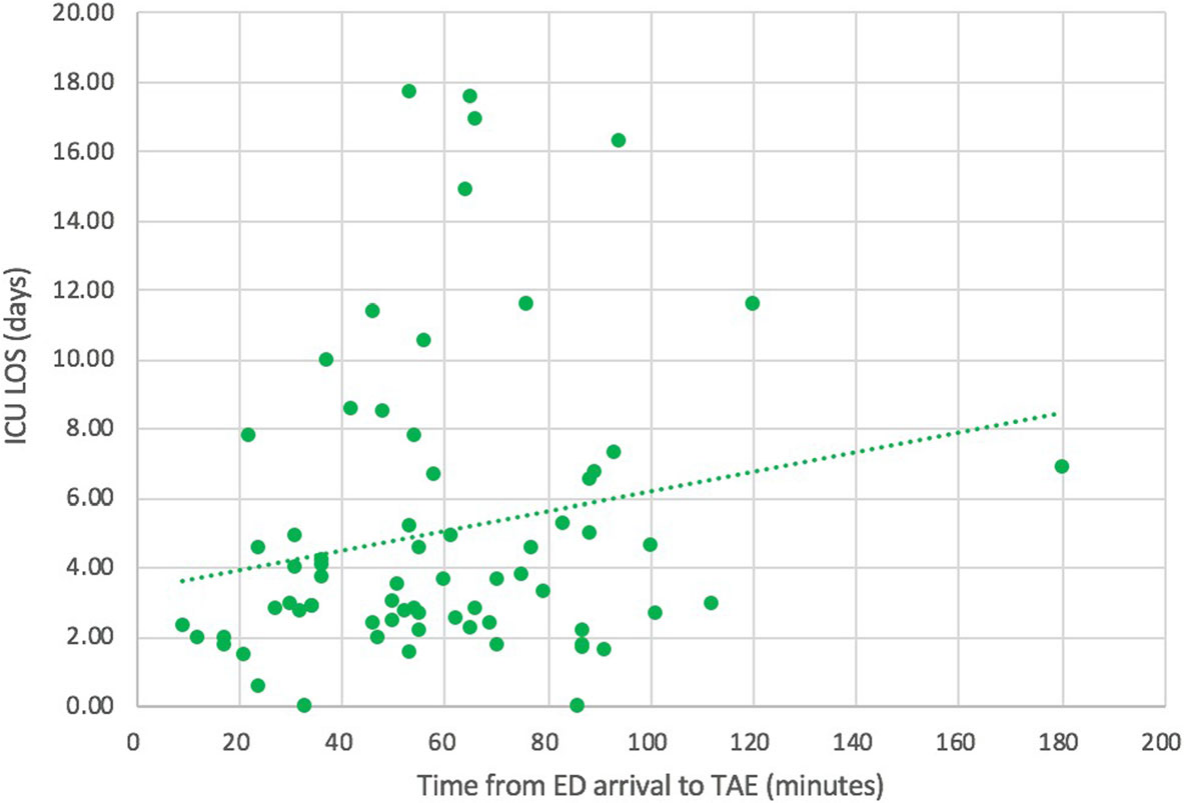
The relationship between the time to TAE and the requirement for blood transfusion

A multivariate logistic regression analysis was performed to evaluate the independent risk factors for mortality. Although AIS of the head/neck and chest were significantly different between the nonsurviving and surviving patients in univariate analysis, AIS was not included in the multivariate logistic regression model due to high multicollinearity between AIS and ISS (the variance inflation factor value was over 10) [29]. After adjustments with physical data, age, and ISS, the time to TAE did not significantly affect mortality, whereas ISS and age served as independent risk factors for mortality. An increase of one unit in ISS or age resulted in 1.154- and 1.140-fold increases in mortality, respectively (*p* = 0.033 and 0.005, respectively; Table 3). In addition, a multivariate linear regression analysis was performed to evaluate the effects of age, SBP, GCS, BD, lactate, ISS, and time to TAE on ICU LOS. The results showed that time to TAE was an independent indicator of ICU LOS (*p* = 0.015; Table 4). Figure 3 also demonstrates the positive relationship between the time to TAE and ICU LOS. In addition, Fig. 4 demonstrates the positive relationship between the time to TAE and the requirement for blood transfusion.

**Table 3:** Logistic regression analysis of the independent risk factors of mortality in pelvic fracture patients who received TAE

*p* value		Odds ratio to mortality	95% CI	
			Lower	Upper
ISS	0.002	1.120	1.044	1.202
Age (y/o)	0.015	1.048	1.009	1.088
Sex (male)	0.384	0.526	0.124	2.235
Time to TAE (min)	0.126	1.015	0.996	1.034

**Table 4:** Linear regression analysis of the factors affecting ICU LOS

*p* value		Unstandardized Coefficients		Standardized Coefficients	T
		B	Std. Error	Beta	
ISS	0.042	0.111	0.054	0.225	2.069
Age (y/o)	0.777	− 0.009	0.031	− 0.31	− 0.284
Time to TAE (min)	0.412	0.016	0.019	0.090	0.824

## Discussion

Pelvic fractures mainly occur as a result of high-energy blunt trauma, such as traffic accidents and falling accidents [1, 2]. Subsequent substantial hemorrhage can significantly increase mortality, and the management of this type of hemorrhage can be challenging [2]. A total of 1339 patients with pelvic fracture were enrolled from 11 level-I trauma centers across the USA, 30 of whom (16.9%) were treated with therapeutic TAE. No consensus is available regarding the optimal treatment paradigm for patients presenting with hemorrhage from severe pelvic fracture, and significant variation exists across institutions [30]. Four options for severe lesions resulting from pelvic trauma have been suggested by the World Society of Emergency Surgery, including pre-peritoneal packing (PPP), temporary mechanical stabilization, resuscitative endovascular balloon occlusion of the aorta (REBOA), and TAE [31]. The recent evolution of these four procedures has significantly decreased the mortality rates of devastating pelvic injuries [32].

Approximately 85% of pelvic fracture hemorrhage results from bone or venous bleeding [2, 10]. In these cases, to control bleeding, temporary mechanical stabilization methods, such as circumferential sheet wrapping and pelvic packing, can be used [11, 33–35]. Nevertheless, arterial bleeding due to pelvic fractures that is not controlled by only mechanical stabilization methods is more troublesome and should be treated more aggressively and promptly [9]. TAE is the most effective intervention for the management of hemorrhage associated with pelvic fracture in both hemodynamically stable and unstable patients and can be used as the primary definitive intervention or in conjunction with operative management in the setting of concomitant intra-abdominal injury [36]. This procedure is increasingly used to control arterial bleeding and is successful in 85% to 100% of cases with regard for bleeding control and reducing the requirement for blood transfusion [5, 10, 14–21]. Cooperative guidelines from Italy showed that after non-pelvic sources of blood loss have been excluded, patients with pelvic fractures and hemodynamic instability or signs of ongoing bleeding should be considered for TAE. Patients with CT scans demonstrating arterial intravenous contrast extravasation in the pelvis may require TAE regardless of hemodynamic status. Furthermore, repeat TAE should be considered in patients with pelvic fracture who have undergone TAE but have persisting signs of ongoing bleeding [37]. For patients with unstable pelvic fractures, early transfer to trauma centers was suggested because of their increasing tendency of requiring TAE [38]. On the other hand, PPP is a more rapid treatment method for severe pelvic trauma than TAE and is suitable for patients with hemodynamic instability at centers without an in-house interventional radiology staff available at all times [39]. PPP may be useful as a bridge for time-consuming procedures, such as TAE [40].

In the current study, we hypothesized that earlier TAE would result in better outcomes. In a previous study, the mortality rate in patients who received TAE less than 3 h after ED admission was 36.4%, whereas the mortality rate was 75% in patients who received TAE more than 3 h after ED admission [19]. Balogh further noted that patients with pelvic fractures and unstable hemodynamics should receive TAE within 90 min after ED admission, as this reduced blood transfusion volumes and mortality [7]. Another retrospective study by Tanizaki et al. reported that in hemodynamically unstable patients with pelvic fractures, between nonsurvivors and survivors, the average time from hospital arrival to angiography was 89.9 ± 28.6 vs. 63.1 ± 23.5 min, respectively [22]. In contrast, a larger case number was included in the current study (84 vs. 24), and we found that there was no significant difference in the time to TAE between nonsurvivors and survivors (76.9 ± 47.9 vs. 59.0 ± 29.3 min, *p* = 0.068). Although an association between mortality and the time to TAE was not observed in the current study. However, it is arbitrary to conclude that the time to TAE did not affect outcomes of pelvic fractures based on the above results. There is still a trend to increased mortality with longer time to TAE, but it does not establish a standard 0.05 *p* value. In our institution, the mean time to TAE was 62.0 ± 33.4 min, indicating that the majority of patients underwent TAE within approximately 90 min after admission. TAE is uniformly urgent and is performed within a very short time-frame; thus, it would be difficult to make an impact in difference in outcomes. It is possible that this difference would be significant in a larger sample size.

In the current study, we further analyzed how the time to TAE affected the requirement for blood transfusion and ICU LOS, and we found that there were trends in which longer waiting times to TAE resulted in both a higher requirement for blood transfusion and longer ICU LOS. Figure 3 demonstrates that there was a positive relationship between the time to TAE and ICU LOS. Table 4 also demonstrates that the time to TAE was an independent indicator of ICU LOS. In addition to ICU LOS, a positive relationship between time to TAE and requirement for blood transfusion was also observed (Fig. 4). Previous studies also reported that delayed hemorrhage was a risk factor for massive blood transfusion, and massive blood transfusion was associated with increased ICU LOS [4, 6, 41–43]. Although a negative effect of the time to TAE on mortality among pelvic fracture patients who required TAE for hemostasis was not observed, a longer waiting time for TAE may increase the morbidity of such patients.

The other concerns are physical data and injury severity of patients with pelvic fractures. We agree that the worse outcomes of pelvic fracture patients who required TAE could be explained simply by longer time to TAE. The roles of poor physical data and more severe overall injuries should also be considered. It is reasonable that older age (odds ratio = 1.140, *p* = 0.005) and higher ISS (odds ratio = 1.154, *p* = 0.033) may result in more mortality (Table 3). Furthermore, lower GCS upon arrival and higher lactate also increase the ICU LOS independently (Table 4). Previous studies also reported that lower GCS, higher lactate level, and higher ISS are associated with massive blood transfusion [44].

The major limitations of this study are that this was a retrospective analysis and that a small patient sample size was drawn from a single institution. Therefore, a type II error was inevitable, which may account for the lack of a statistically significant association between the time to TAE and outcomes. In addition, this study only included a 3-year follow-up and therefore lacks long-term follow-up, which may also have influenced our results. Therefore, a prospective study with a larger patient sample size should be designed to analyze the relationship between the time to TAE and patient outcomes.

## Conclusion

In pelvic fracture patients who require TAE for hemostasis, longer time to TAE may cause harm. An early hemorrhage control is suggested.

## Abbreviations

AIS: Abbreviated injury scale
ATLS: Advanced Trauma Life Support
BD: Base deficit
ED: Emergency department
EIA: External iliac artery
GCS: Glasgow Coma Scale
ICU: Intensive care unit
IIA: Internal iliac artery
ISS: Injury severity score
LOS: Length of stay
OHCA: Out-of-hospital cardiac arrest
SBP: Systolic blood pressure
TAE: Transcatheter arterial embolization
